# Inhibition of CT26 Colon Cancer Cell Growth by Green-Synthesised Silver Nanoparticles-Mediated *Strobilanthes crispus* Extract

**DOI:** 10.21315/mjms-11-2025-798

**Published:** 2026-08-31

**Authors:** Siti Aisyah Abd Ghafar, Ariff Haikal Hairil Anuar, Muhammad Ariff Hakimi Salihuddin, Rohazila Mohamad Hanafiah

**Affiliations:** Department of Basic Sciences, Faculty of Dentistry, Universiti Sains Islam, Malaysia, Pandan Indah, 55100 Ampang, Kuala Lumpur, Malaysia

**Keywords:** Strobilanthes crispus, silver nanoparticles, CT26 colon carcinoma, apoptosis, cell cycle arrest, green synthesis

## Abstract

**Background:**

This study evaluated the antiproliferative and pro-apoptotic activities of green-synthesised silver nanoparticles using *Strobilanthes crispus* extract (AgNP-SC) against CT26 colon carcinoma cells. *Strobilanthes crispus* (*S. crispus*), rich in phytochemicals, was utilised as a natural reducing and stabilising agent during AgNP synthesis, potentially enhancing their cytotoxic potential.

**Methods:**

AgNP-SC was synthesised by reducing silver nitrate with an aqueous extract of *S. crispus*, with nanoparticle formation indicated by the appearance of a dark brown colour. Cytotoxicity was assessed using the 3-(4,5-dimethylthiazol-2-yl)-2,5-diphenyltetrazolium bromide (MTT) assay. Apoptotic changes were examined through acridine orange/propidium iodide (AO/PI) staining, while cell cycle distribution was analysed using flow cytometry. Gene expression of apoptotic markers was analysed using reverse transcription polymerase chain reaction (RT-PCR).

**Results:**

MTT analysis demonstrated a significant, concentration-dependent decrease in CT26 cell viability following AgNP-SC treatment, with cell viability reduced to below 50% at concentrations ≥ 25 μg/mL (*P* < 0.05). AO/PI staining revealed classical apoptotic features, including chromatin condensation, membrane blebbing, and nuclear fragmentation. Flow cytometric analysis showed a significant accumulation of cells in the G_1_ phase accompanied by a marked reduction in the S-phase population in treated groups compared to untreated controls (*P* < 0.05), indicating G_1_ cell cycle arrest and inhibition of DNA synthesis. RT-PCR analysis further demonstrated significant upregulation of pro-apoptotic genes (*CED-3* and *CED-4*) and downregulation of the anti-apoptotic gene *CED-9* (*P* < 0.05), confirming activation of the intrinsic mitochondrial apoptotic pathway.

**Conclusion:**

Green-synthesised AgNP-SC exhibits potent anticancer properties, suggesting its potential as a natural-based nanotherapeutic agent for colon cancer. Further *in vivo* and mechanistic studies are warranted to validate its therapeutic efficacy.

## Introduction

Colon cancer, also known as bowel cancer or colorectal cancer, is characterised by the uncontrollable growth of cells in the colon or rectum, which are parts of the large intestine. It is among the most frequently diagnosed cancers worldwide (1). In 2020, approximately 1,931,590 new colorectal cancer cases were reported worldwide, with males accounting for 55.18% of all cases (2). Globally, the burden of early-onset colorectal cancer (EOCRC) increased between 1990 and 2021. Incidence rose from 5.43 to 6.13 per 100,000 population, and prevalence increased from 29.65 to 38.86 per 100,000 population. In contrast, mortality declined from 2.98 to 2.30 per 100,000 population, and disability-adjusted life years decreased from 148.46 to 115.42 per 100,000 population. In 2021, East Asia and China had the highest EOCRC burden at the regional and national levels, respectively. Population growth was the main factor contributing to the increased burden. Countries with a high sociodemographic index experienced a greater EOCRC burden than those with a low sociodemographic index. The global incidence and prevalence of EOCRC are projected to continue rising from 2022 to 2030 (3). In Malaysia, most patients with colon cancer are diagnosed at an advanced stage, resulting in a poor prognosis, reduced quality of life, and increased treatment costs. Current treatment options, including surgery, chemotherapy, radiotherapy, and targeted therapies, are associated with several limitations. Consequently, there is a growing interest in developing safer and more effective anticancer therapies. Therefore, the present study aimed to investigate the antiproliferative and proapoptotic effects of green-synthesised silver nanoparticles-*Strobilanthes crispus* (AgNP-SC) against CT26 colon carcinoma cells by evaluating cytotoxicity, apoptosis, cell cycle progression, and apoptosis-related gene expression.

Although numerous studies have reported the anticancer potential of green-synthesised AgNPs, most investigations have focused on human cancer cell lines and have primarily described cytotoxic effects without detailed mechanistic validation (4). In particular, limited data are available on the molecular mechanisms underlying apoptosis and cell cycle regulation induced by plant-mediated AgNPs in immunocompetent colorectal cancer models.

The murine CT26 colon carcinoma cell line is a biologically relevant model for colorectal cancer because of its syngeneic compatibility with BALB/c mice, enabling direct translation to *in vivo* and immunological studies. However, the cytotoxic and apoptotic mechanisms of green-synthesised AgNPs in CT26 cells remain poorly characterised despite the widespread use of this model in preclinical colon cancer research (5).

*Strobilanthes crispus* (*S. crispus*) is rich in bioactive flavonoids and phenolic compounds with reported anticancer properties (6, 7). However, its application as a reducing and stabilising agent in AgNP synthesis for colon cancer therapy has not been mechanistically explored (8). A previous study demonstrated that AgNP-SC exhibited potent antibacterial activity against *Streptococcus mutans*, *Pseudomonas aeruginosa* and *Escherichia coli* (6). The study also characterised the synthesised AgNP-SC. AgNP-SC exhibited a spherical morphology with an average particle size of 75.25 nm. Therefore, the present study was designed to investigate the antiproliferative and pro-apoptotic effects of AgNP-SC in CT26 colon carcinoma cells.

## Methods

### Preparation of AgNP-SC

In this study, *S. crispus* extract was prepared following a previously described method (6). The leaves were washed and dried in an oven at 45 °C. The dried leaves were then ground into a fine powder and soaked in distilled water at 60 °C for 24 h. The aqueous extract was filtered through Whatman No. 1 filter paper and freeze-dried to obtain *S. crispus* extract (Figure 1). *S. crispus* leaves were collected in June 2023.

To synthesise AgNP-SC, silver nitrate was mixed with *S. crispus* extracts and stirred continuously overnight. Colour changes to reddish-brown indicated the formation of AgNP-SC. The solution was then freeze-dried to obtain AgNP-SC powder. Approximately 26% AgNP-SC yield was obtained by mixing 450 mg of *S. crispus* powder with 153 mg of silver nitrate in 1 L of water.

### Culture of CT26 Colon Carcinoma Cells

CT26 colon carcinoma cells were cultured in Roswell Park Memorial Institute (RPMI)-1640 medium supplemented with 10 % fetal bovine serum (Nacalai Tesque, Japan) and 1% penicillin-streptomycin (Nacalai Tesque, Japan). The cells were incubated at 37 °C in a humidified incubator containing 5% CO_2._

### Cell Viability Assay

The CT26 colon carcinoma cells were seeded in 96-well plates at a density of 1 × 10^5^ per well. Cells were incubated at 37 °C in a humidified incubator containing 5% CO_2_ for 24 h. After that, cultured cells were treated with AgNP-SC at concentrations ranging from 3.125 to 200 μg/ml, in triplicate, and incubated for 24 h. Subsequently, 3-(4,5-dimethylthiazol-2-yl)-2,5- diphenyltetrazolium bromide (MTT) (Sigma, St. Louis, MO, USA) solution (5 mg/mL) was added to each well, followed by incubation for another 4 h. The medium was removed, and formazan was solubilised with dimethyl sulphoxide (DMSO) (Sigma, St. Louis, MO, USA). The absorbance of the solution was measured at 570 nm using a SpectraMax iD3 microplate reader (Molecular Devices, USA). Half-maximal inhibitory concentration (IC_50_), 25% inhibitory concentration (IC_25_), and 75% inhibitory concentration (IC_75_) were determined from the plotted graph by converting the absorbance value to percentage cell viability. The IC_50_ value was determined manually by plotting lines at 50% cell viability on the graph. All experiments were performed in triplicate.

### Morphological Changes of CT26 Colon Carcinoma Cells

CT26 colon carcinoma cells were seeded at a density of 5 × 10^4^ cells/well in 6-well plates and incubated for 24 h. The cells were treated with IC_25_ and IC_50_ concentration values. Cells were then trypsinised, centrifuged and washed once with phosphate-buffered saline (PBS). The pellets were centrifuged again, and the remaining pellets were mixed with 5 μL of acridine orange (AO) and 5 μ L of propidium iodide (PI) (Sigma, USA), followed by observation under a fluorescence microscope (Olympus, Germany). All experiments were performed in triplicate.

### Cell Cycle Distribution Analysis

The procedure was adapted from a previous study with some modifications (9). Cells were seeded into 6-well plates at a density of 1 × 10_6_ cells/well and treated with AgNP-SC at concentrations corresponding to the IC_50_, IC_60_, and IC_75_ values. After treatment, cells were collected by centrifugation and washed twice with 5 mL of PBS. The cell pellet was fixed with 5 mL of cold 70% ethanol at –20 °C for 2 h. Subsequently, the ethanol was removed, and the cells were washed with PBS and centrifuged. The pellet was then resuspended in 425 μL of PBS containing 5 μL of ribonuclease A (RNase A) (100 mg/mL) and 20 μL of propidium iodide (PI, 1 mg/mL) and incubated in the dark at 4 °C for 30 min. Approximately 100,000 stained cells were analysed using a flow cytometer (Becton Dickinson, USA). All experiments were performed in triplicate.

### Ribonucleic Acid Extraction

Ribonucleic Acid (RNA) was extracted using the SV Total RNA Isolation System (Promega, Wisconsin, USA) according to the manufacturer’s protocol. RNA was extracted from CT26 colon carcinoma cells treated with AgNP-SC at IC_25_, IC_50_, and IC_75_ concentrations, as well as from untreated control cells. After purification, RNA concentration was measured using a NanoDrop 2000c spectrophotometer (Thermo Fisher Scientific, MA, USA). The A260/A230 ratio was checked to ensure the RNA was free from contaminants such as proteins, phenol, and salts. All samples were analysed in triplicate.

### Real-Time Polymerase Chain Reaction (RT-PCR)

Real-time polymerase chain reaction (RT-PCR) analysis was performed using SensiFAST™ SYBR® No-ROX One-Step Kit (Bioline, UK). The primers used for *CED-9* (Forward: 5′-GTTACTCCAGCGCCAATCAT-3′; Reverse: 5′-CAAGTGCGAAACCTCTTCGT-3′), *CED-4* (Forward: 5′-CGAAAGAGCTTGTGATGCAA-3′; Reverse: 5′-CAATTTTCCCGACAAATGCT-3′), *CED-3* (Forward: 5′-TCAGCAGCTCAACAA ACATCC-3′; Reverse: 5′-GCAAGTTCAGCAA GTGTGTGGA-3′), and glyceraldehyde-3- phosphate dehydrogenase (*GAPDH*) (Forward: 5′-CAAGGTCATCCATGACAACTTTG-3′; Reverse: 5′-GTCCACCACCCTGTTGCTGTAG-3′).

Glyceraldehyde-3-phosphate dehydrogenase (GAPDH) (F-CAAGGT CATCCATGACAACTTTG, R-GTCCACCACCCTG TTGCTGTAG) was used as the housekeeping gene. An RT-PCR assay was performed using the CFX96 Touch Real-Time PCR Detection System (Bio-Rad Laboratories, Hercules, CA, USA). Reaction mixtures were prepared according to the manufacturer’s protocol. The amplification programme consisted of 50 °C for 10 min and 95 °C for 10 min, followed by 35 cycles of 95 °C for 15 s and 60 °C for 1 min. No-template RNA was used as the negative control. Gene expression was normalised using the 16S ribosomal RNA (16S rRNA) reference gene. Relative gene expression was calculated using the (2^−ΔΔCt^) method described by Livak and Schmittgen (10). All experiments were performed in triplicate.

## Results

### Preparation and Characterisation of AgNP-SC

Upon mixing the aqueous *S. crispus* extract with the silver nitrate solution, a gradual colour change to dark brown was observed, indicating the synthesis of AgNP-SC. Following freeze-drying, a brown powder with a distinct pungent odour was obtained (11). Green-synthesised silver nanoparticles derived from *S. crispus* (AgNP-SC) were successfully produced using an aqueous plant extract and silver nitrate. Formation of AgNP-SC was confirmed by ultraviolet-visible (UV–Vis) spectroscopy, which exhibited a characteristic surface plasmon resonance peak at approximately 400 nm. Previous physicochemical characterisation demonstrated that AgNP-SC had an average particle size of 75.25 nm, a polydispersity index (PDI) of 0.373, and a zeta potential of −35.6 mV (6). Field emission scanning electron microscopy (FESEM) analysis revealed predominantly spherical nanoparticles with a rough surface morphology, while X-ray diffraction analysis confirmed the crystalline nature (6).

### Cytotoxicity of AgNP-SC

The MTT assay was performed to evaluate the cytotoxic effects of AgNP-SC against CT26 colon carcinoma cells by determining the half-maximal inhibitory concentration (IC_50_). Each independent trial consistently yielded an IC_50_ value of 15 μg/mL for AgNP-SC, demonstrating its cytotoxic activity against CT26 cells (Figure 2a). Statistical analysis revealed a significant difference (*P* < 0.05) in cell viability between treated and control groups. Results from the triplicate assays were pooled, yielding an average IC_50_ value of 15 μg/mL. For CT26 colon carcinoma cells, AgNP-SC induced a sharp reduction in cell viability even at low concentrations. Cell viability dropped markedly below 50% at approximately 25 μg/mL and further decreased to below 10% at concentrations ≥ 50 μg/mL. At higher concentrations (100 to 200 μg/mL), CT26 cell viability remained consistently low, indicating a strong and sustained cytotoxic effect. These results indicate that AgNP-SC exerts potent cytotoxic effects against CT26 colon carcinoma cells in a concentration-dependent manner.

For comparison, CT26 colon carcinoma cells were treated with 5-fluorouracil (5-FU) and *S. crispus* aqueous extract, which served as the positive and negative controls, respectively. The average IC_50_ value of 5-FU was 12 μg/mL, whereas no measurable IC_50_ was observed for the *S. crispus* aqueous extract (Figure 2b). The cytotoxic activity of AgNP-SC was further evaluated in CT26 colon carcinoma cells and compared with that in 3T3 normal fibroblast cells over a concentration range of 0 to 200 μg/mL. AgNP-SC reduced the viability of CT26 cells in a concentration-dependent manner.

In contrast, 3T3 normal fibroblast cells maintained relatively high viability across all tested concentrations (Figure 2c). Although cell viability decreased slightly with increasing AgNP-SC concentration, it remained above approximately 70% even at the highest concentration tested (200 μg/mL).

A significant difference (*P* < 0.05) in cell viability between CT26 and 3T3 cells at corresponding concentrations demonstrated the selective cytotoxicity of AgNP-SC. Such selectivity is a critical characteristic of a promising anticancer agent, as it suggests the ability to preferentially target malignant cells while sparing normal healthy cells.

Overall, these findings demonstrate that AgNP-SC possesses potent anticancer activity with good biocompatibility, supporting its potential as a therapeutic candidate for colon cancer treatment. Further mechanistic studies, including apoptosis assays, reactive oxygen species generation analysis, and *in vivo* evaluations, are warranted to elucidate the underlying pathways and confirm its safety and efficacy.

The cytotoxic effect of *S. crispus* extract on CT26 colon carcinoma cells was evaluated over a concentration range of 0 to 200 μg/mL. As shown in Figure 2d, cell viability remained consistently high across all tested concentrations, generally ranging from 80% to 90%. At lower concentrations, a slight reduction in cell viability was observed; however, this effect was minimal and did not show a clear dose-dependent trend. Even at the highest concentration tested (200 μg/mL), CT26 cell viability remained close to that of the untreated control, indicating that the *S. crispus* extract alone exerted limited cytotoxic activity against CT26 cells.

The absence of a pronounced dose-dependent decrease in cell viability suggests that the crude aqueous extract of *S. crispus* does not exhibit strong anticancer effects under the conditions tested. Compared with the marked cytotoxicity observed for AgNP-SC in CT26 cells, these results highlight that nanoparticle formulation significantly enhances the anticancer efficacy of *S. crispus*. The enhanced activity of AgNP-SC may be due to improved cellular uptake, increased surface reactivity, and sustained interaction with intracellular targets.

Overall, these findings indicate that while *S. crispus* extract alone shows minimal cytotoxic effects on CT26 colon cancer cells, its incorporation into silver nanoparticles substantially improves its anticancer potential.

Silver nanoparticles (AgNPs) exhibit cytotoxic effects in cancer cells via multiple pathways, encompassing the generation of reactive oxygen species, the initiation of apoptosis, and the disruption of cell cycle progression. Furthermore, their potential for targeted delivery offers a means to enhance therapeutic efficacy while mitigating adverse off-target effects.

### Morphological Changes of CT26 Colon Carcinoma Cells

Morphological changes in CT26 colon carcinoma cells following AgNP-SC treatment were evaluated using acridine orange/propidium iodide (AO/PI) staining. Untreated cells predominantly exhibited green fluorescence, indicating viable and healthy cells. In contrast, AgNP-SC-treated cells displayed both green and red fluorescence, indicating the presence of viable as well as damaged or non-viable cells. Cells treated with AgNP-SC exhibited morphological features consistent with apoptosis, including chromatin condensation, cytoplasmic shrinkage, membrane blebbing, and nuclear fragmentation. Cells treated with the IC_25_ concentration showed a reduction in overall cell number and an increase in red fluorescent signals, indicative of extensive cell death.

Untreated control cells displayed characteristic features of healthy morphology, including intact cell shape and smooth cell surfaces. AgNP-SC-treated cells exhibited more pronounced morphological alterations, including cell shrinkage, disruption of cell-cell contacts, and increased chromatin condensation. Treatment at IC_50_ concentration intensified these effects, with additional features including pronounced cell shrinkage, disruption of cell-cell contacts, and further chromatin condensation. Figure 3 illustrates these morphological changes in greater detail.

The AO/PI staining results demonstrated concentration-dependent morphological changes in CT26 colon carcinoma cells following AgNP-SC treatment. Untreated cells retained normal morphology, whereas treated cells exhibited morphological features consistent with apoptosis, including chromatin condensation, membrane blebbing, cytoplasmic shrinkage, and nuclear fragmentation. These findings suggest that AgNP-SC induces apoptotic cell death in CT26 colon carcinoma cells.

### Cell Cycle Distribution

Flow cytometric analysis was performed to evaluate the effect of AgNP-SC treatment on CT26 colon carcinoma cell cycle progression and to determine the phase at which cell cycle arrest occurred. Analysis of untreated and AgNP-SC–treated CT26 cells at different concentrations revealed no significant difference (*P* > 0.05) in the proportion of cells in the G_1_ phase between the control and treated groups. However, all treated groups displayed a significantly lower percentage of cells in the S phase than the control group (*P* < 0.05). In the G_2_ phase, significant differences (*P* < 0.05) were observed in the IC_60_ and IC_50_ groups relative to the control. Cell cycle analysis demonstrated distinct differences between untreated and AgNP-SC-treated CT26 colon carcinoma cells. The untreated control exhibited normal cell cycle progression, whereas treated cells showed altered cell cycle distribution. In contrast, AgNP-SC- treated cells exhibited cell cycle arrest at the G_1_ phase. This was accompanied by a reduction in the proportion of cells in S phase compared with the untreated control. Quantitative analysis of cell cycle distribution is presented in Figure 4.

The observed G_1_ phase arrest suggests that AgNP-SC affects cell cycle progression in CT26 colon carcinoma cells. Cell cycle regulators regulate cell growth. In the present study, AgNP-SC induced cell cycle arrest in the G_1_ phase, indicating that it affected cell cycle regulators (12–14). G_0_–G_1_ transcription was regulated by the expression of G0S2, c-jun, CSB, KU and NBS1 (15, 16). The cell cycle was primarily driven by cyclin-dependent kinases (CDKs) and cyclins (17). The complexes including cyclin D– CDK4/6 and cyclin E–CDK2 drive the transition from G_1_ to S phase. The cyclin D–CDK4/6 complex was essential for entering G_1_ phase, while the cyclin E–CDK2 complex regulates the induction of DNA synthesis in late G_1_ and early S phases (18). The INK4 proteins specifically interfere with the association between cyclin D and CDK4/6, whereas the Cip/Kip family of inhibitors such as p21 and p27 inhibit cyclin E– CDK2 activity (19).

### Gene Expression

The RT-PCR analysis was performed to evaluate changes in gene expression and to provide insight into the mechanism by which AgNP-SC induces apoptosis in CT26 colon carcinoma cells. RT-PCR analysis showed differences in the expression of apoptosis-related genes in CT26 colon carcinoma cells following AgNP-SC treatment. The genes selected for observation were *CED-3*, *CED-4*, and *CED-9*. These are the main genes that are responsible for the apoptosis process in cells. *CED-3* and *CED-4* are pro-apoptotic genes which are responsible for activating the apoptosis process and causing cell death. *CED-9* is an anti-apoptotic gene which is responsible for preventing apoptosis from happening and maintaining the survival of the cells. The RT-PCR result showed that the *CED-3* and *CED-4* genes were upregulated while the *CED-9* gene was downregulated. The changes in gene expression were also significantly different (*P* < 0.05) between the untreated group and treated groups. This indicated that apoptosis was happening in the CT26 colon carcinoma cells treated with AgNP-SC, disrupting the proliferation of the cells and leading to the cells’ death. Figure 5 shows the relative expression of *CED-3*, *CED-4*, and *CED-9* genes in CT26 colon carcinoma after AgNP-SC treatment.

In this study, the CT26 colon carcinoma cells treated with AgNP-SC showed upregulation of *CED-3* and *CED-4* genes, and downregulation of *CED-9* gene. *CED-3* is a cysteine-dependent aspartate-specific protease or caspase; *CED-9* is a homolog of the mammalian anti-apoptotic protein Bcl-2, having mammalian counterparts; and *CED-4* can further stimulate the processing of *CED-3* in the cell apoptosis process (20). In living cells, abnormal *CED-4* dimers involved in cell death are sequestered by *CED-9* on the outer surface of the mitochondria and maintained in an inactive conformation. Cells that are destined to die by apoptosis produce the *BH3*-domain protein egg-laying defective *EGL-1*. Binding of pro-apoptotic *EGL-1* to *CED-9* induces a conformational change in the latter, resulting in the release of the *CED-4* dimer from the *CED-9–CED-4* complex. Once freed from the inhibitory interaction with *CED-9*, two *CED-4* dimers associate into a tetramer and recruit *proCED-3* molecules to form the so-called apoptosome. *CED-3* becomes activated (through conformational change and/or proteolysis), and apoptosis is triggered (20).

## Discussion

The present study demonstrated the successful synthesis of green-synthesised AgNP-SC and evaluated their anticancer activity against CT26 colon carcinoma cells. The findings showed that AgNP-SC exhibited potent antiproliferative activity, induced apoptotic morphological changes, altered cell cycle progression, and modulated the expression of apoptosis-related genes. Collectively, these results suggest that AgNP-SC possesses promising potential as a natural-based nanotherapeutic agent for colon cancer.

The aqueous extract of *S. crispus* was combined with silver nitrate to produce AgNP-SC. UV–Vis spectroscopy confirmed the formation of AgNP-SC, showing a characteristic peak at approximately 400 nm. According to a previous study, AgNP-SC had an average particle size of 75.25 nm, a polydispersity index (PDI) of 0.373, and a zeta potential of –35.6 mV, as measured using a Zetasizer. FESEM analysis revealed spherical nanoparticles with a rough surface morphology, while X-ray diffraction confirmed their crystalline structure (6). AgNP-SC also exhibited potent antiproliferative activity against CT26 colon carcinoma cells, with an IC_50_ of 15 μg/mL. This finding suggests that AgNP-SC possesses strong cytotoxic potential comparable to the standard chemotherapeutic agent 5-fluorouracil (5-FU), which exhibited an IC_50_ of 12 μg/mL. Importantly, the aqueous extract of *S. crispus* alone did not produce measurable cytotoxicity, indicating that incorporating the plant’s bioactive compounds into silver nanoparticles may play a critical role in enhancing anticancer activity.

The observed cytotoxicity of AgNP-SC may result from synergistic interactions between the phytoconstituents of *S. crispus*, including flavonoids, phenolic acids, and catechins and the inherent physicochemical properties of silver nanoparticles. These phytochemicals act as both reducing and stabilising agents during nanoparticle synthesis, producing biocompatible particles with enhanced cellular uptake and reactive oxygen species generation (20). Elevated reactive oxygen species levels disrupt mitochondrial integrity, induce oxidative stress, and activate intrinsic apoptotic pathways, ultimately leading to controlled cell death (21).

Cellular stress induced by nutrient deprivation or exposure to cytotoxic agents can trigger either apoptotic or necrotic pathways of cell death. AO/PI staining was a widely used method to distinguish between these types. AO permeates intact membranes and stains viable cells green, while PI penetrates only damaged membranes, staining necrotic or late apoptotic cells red. Apoptotic cells typically appear shrunken and green with condensed nuclei, whereas necrotic cells emit red fluorescence. Microscopic observations using AO/PI staining confirmed that AgNP-SC treatment induced morphological features characteristic of apoptosis, including chromatin condensation, nuclear fragmentation, and membrane blebbing. These hallmarks of apoptosis, rather than necrosis, suggest that AgNP-SC induces programmed cell death, which is beneficial for therapeutic applications (12, 13). Induction of apoptosis is desirable in cancer therapy because it enables the selective elimination of malignant cells while minimising inflammation and collateral tissue damage. Conversely, necrosis is an uncontrolled, pathological process. These two forms of cell death are not mutually exclusive and may co-occur within the same tissue, particularly when cells are exposed to varying concentrations of cytotoxic agents (14).

Flow cytometric analysis revealed that AgNP-SC caused cell cycle arrest predominantly at the G_1_ phase, accompanied by a significant reduction in the proportion of cells in the S phase. This disruption suggests interference with DNA synthesis and checkpoint regulation, potentially through modulation of CDKs and their associated cyclins. The G_1_/S checkpoint is a critical control point ensuring DNA integrity, and its disruption prevents replication of damaged cells (19). Similar G_1_ phase arrest has been reported in cancer cells exposed to biogenic AgNPs, including those synthesised using *Azadirachta indica* and *Camellia sinensis* extracts (22). Thus, AgNP-SC may exert a cytostatic effect by regulating the activity of cyclin D-CDK4/6 and cyclin E-CDK2 complexes, thereby suppressing uncontrolled cell proliferation.

Gene expression analysis by RT-PCR further confirmed the activation of apoptotic pathways. The pro-apoptotic genes *CED-3* and *CED-4* were significantly upregulated, whereas the anti-apoptotic gene *CED-9* was downregulated following AgNP-SC treatment. These findings parallel the mammalian apoptotic pathway involving caspase-3 (homologous to *CED-3*), Apaf-1 (*CED-4*), and Bcl-2 (*CED-9*), in which suppression of Bcl-2 relieves inhibition of the apoptotic complex, allowing caspase activation (23). The observed changes in gene expression suggest that AgNP-SC induces mitochondrial-mediated apoptosis, a mechanism that has also been reported for other greensynthesised silver nanoparticles (24, 25).

The pronounced efficacy of AgNP-SC may be attributed to the phytochemical richness of *S. crispus*, which contains various antioxidant and anticancer compounds (7). Previous studies have demonstrated that *S. crispus* extracts inhibit tumour growth through modulation of phosphatidylinositol 3-kinase/protein kinase B (PI3K/Akt) and mitogen-activated protein kinase signalling pathways (8). Therefore, it is plausible that AgNP-SC enhances these bioactivities through the nanoscale delivery of active compounds, resulting in enhanced cellular stress and apoptosis induction.

Despite these promising findings, this study has several limitations. The selectivity of AgNP-SC towards normal colon epithelial cells was not assessed, and protein-level analyses such as western blotting for caspase activation were not performed. Additionally, *in vivo* studies are necessary to evaluate the biodistribution, pharmacokinetics, and systemic toxicity of AgNP-SC before potential clinical application (26, 27). Future studies should address these limitations to further evaluate the therapeutic safety and efficacy of this nanomaterial.

Overall, the findings indicate that AgNP-SC exerts strong antiproliferative and proapoptotic effects against CT26 colon carcinoma cells through G_1_ phase arrest and mitochondrial-mediated apoptosis. These results highlight the potential of *S. crispus*-based green-synthesised silver nanoparticles as a promising and eco-friendly approach for colon cancer therapy.

## Conclusion

This study demonstrated that silver nanoparticles green-synthesised using an aqueous extract of *S. crispus* (AgNP-SC) exert significant anticancer effects against CT26 colon carcinoma cells. AgNP-SC exhibited potent antiproliferative activity with an IC_50_ of 15 μg/mL, comparable to that of 5-Fluorouracil, and induced apoptosis while inhibiting proliferation through G_1_ phase cell cycle arrest. At the molecular level, AgNP-SC upregulated the pro-apoptotic genes *CED-3* and *CED- 4* while downregulating the anti-apoptotic gene *CED-9*, supporting the activation of the intrinsic apoptotic pathway. Overall, the present findings demonstrate that AgNP-SC exerts potent antiproliferative and pro-apoptotic effects against CT26 colon carcinoma cells, likely through induction of G_1_ phase arrest and activation of the mitochondrial apoptotic pathway. Although further mechanistic and in vivo investigations are required, these results support the potential of *S. crispus*-derived green-synthesised silver nanoparticles as a promising, environmentally sustainable nanotherapeutic strategy for colorectal cancer treatment.

## Figures and Tables

**Figure 1:**
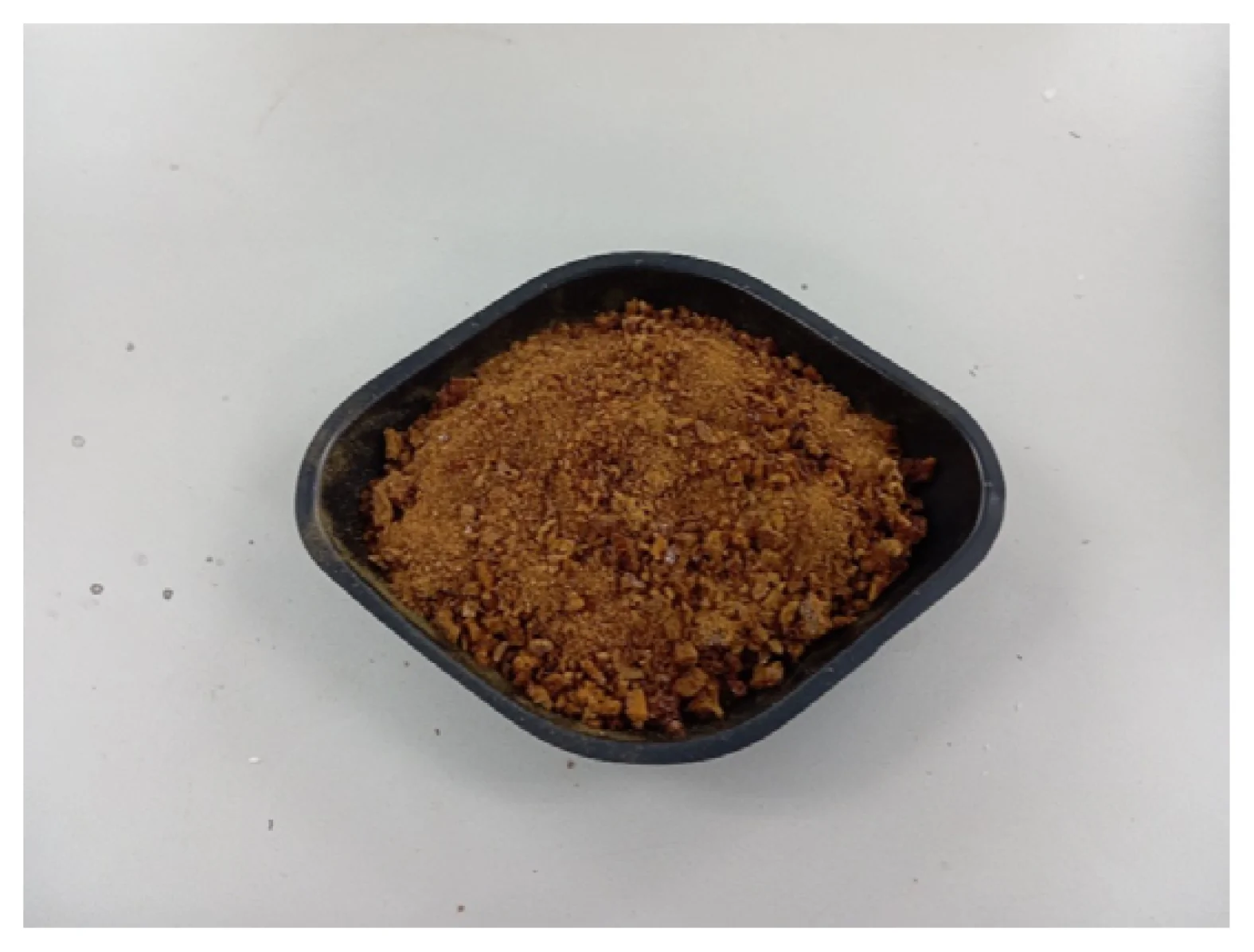
Freeze-dried *S. crispus* aqueous extract. A brown powder with a pungent odour was obtained as the final product.

**Figure 2:**
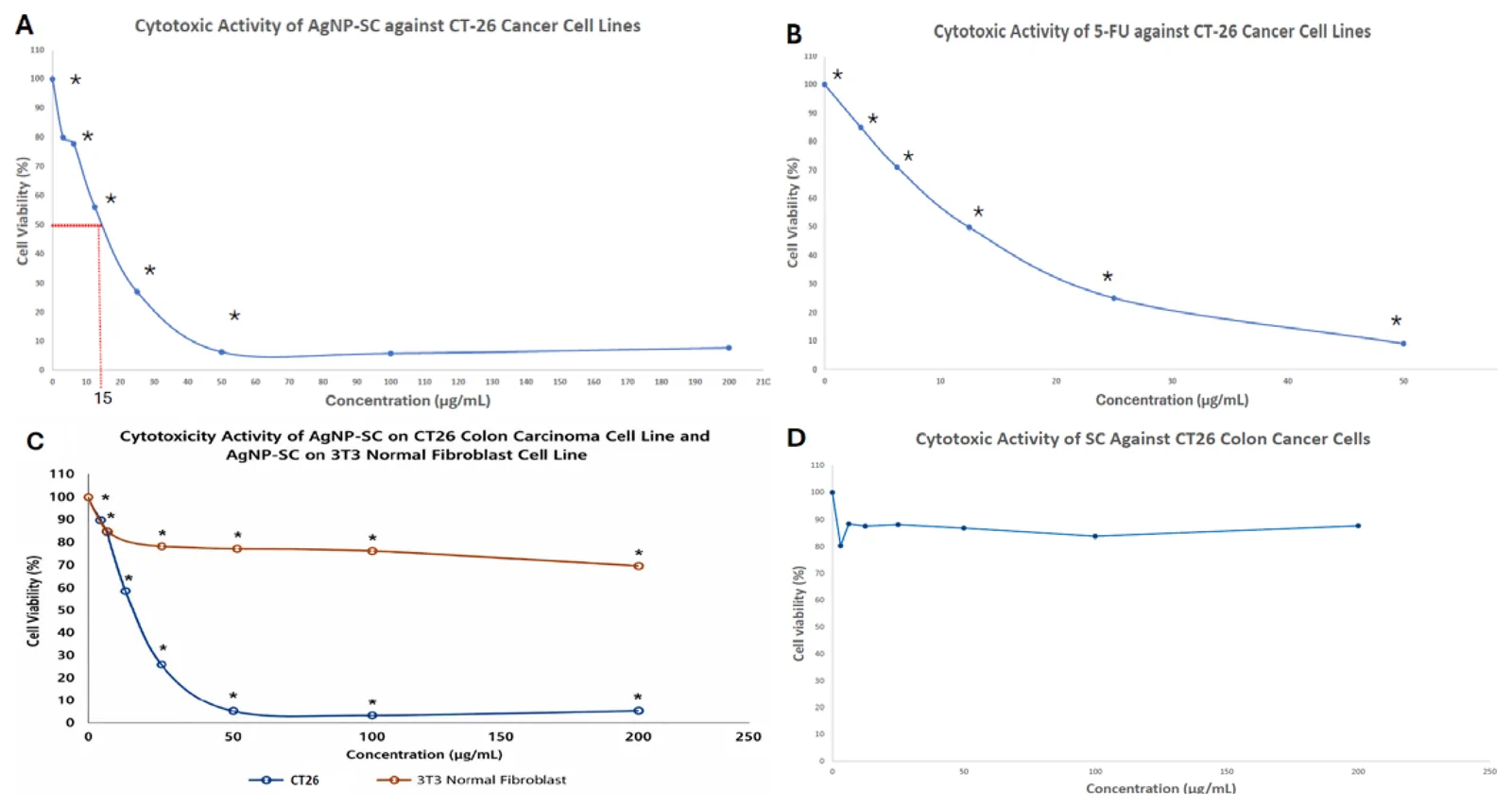
Cytotoxic effects of (a) AgNP-SC, (b) 5-fluorouracil (5-FU), and (c) AgNP-SC on 3T3 normal fibroblast cells, and (d) *S. crispus* aqueous extract on CT26 colon carcinoma cells. The red values indicate the IC_50_ concentrations. Data are presented as mean ± SD (*n* = 3). *P* < 0.05 versus the untreated control.

**Figure 3:**
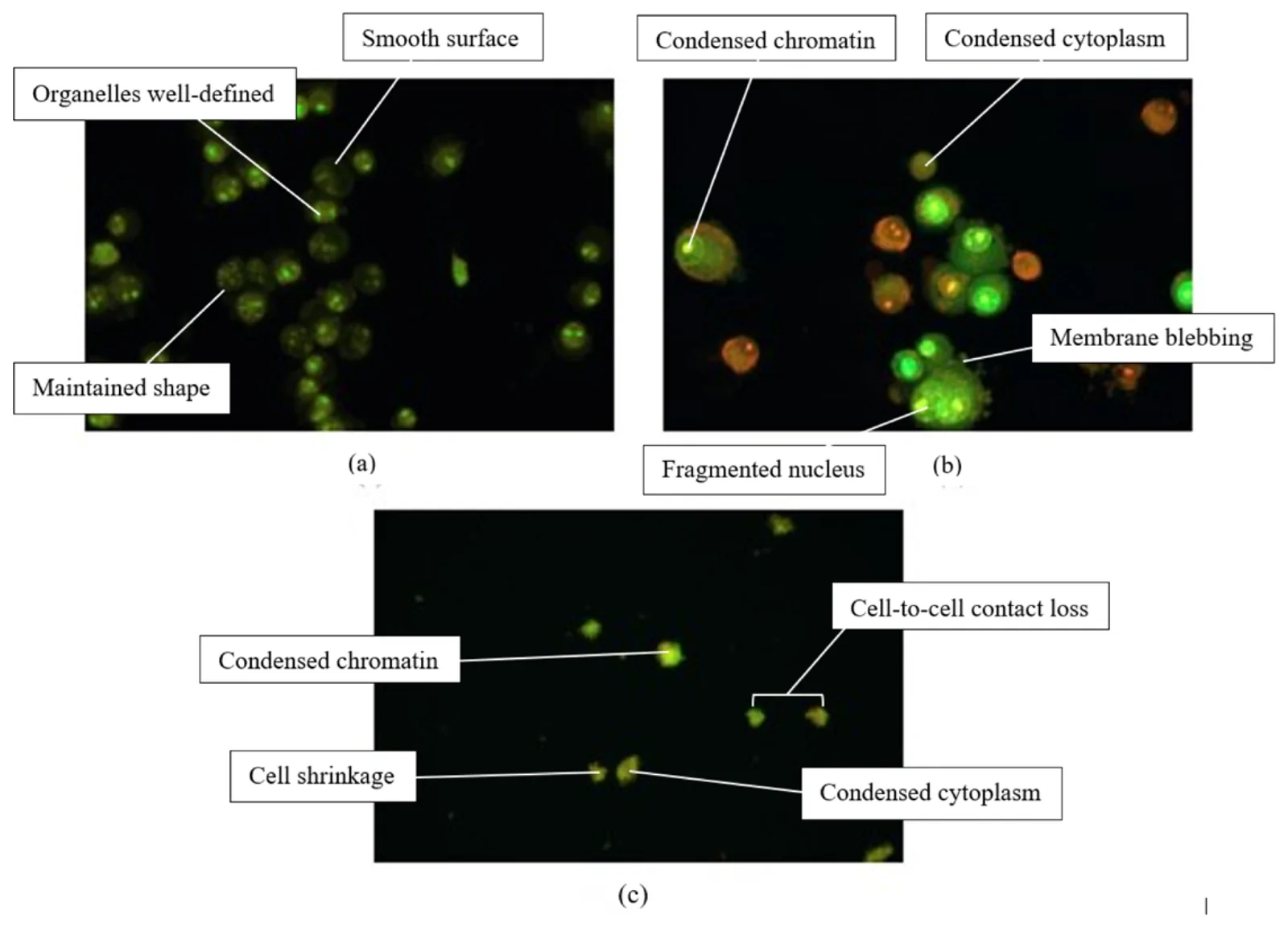
Observation of CT26 colon carcinoma cells under a fluorescence microscope with 40× magnification: (a) untreated cells, (b) cells treated with IC_25_ concentration of AgNP-SC and (c) cells treated with IC_50_ concentration of AgNP-SC.

**Figure 4:**
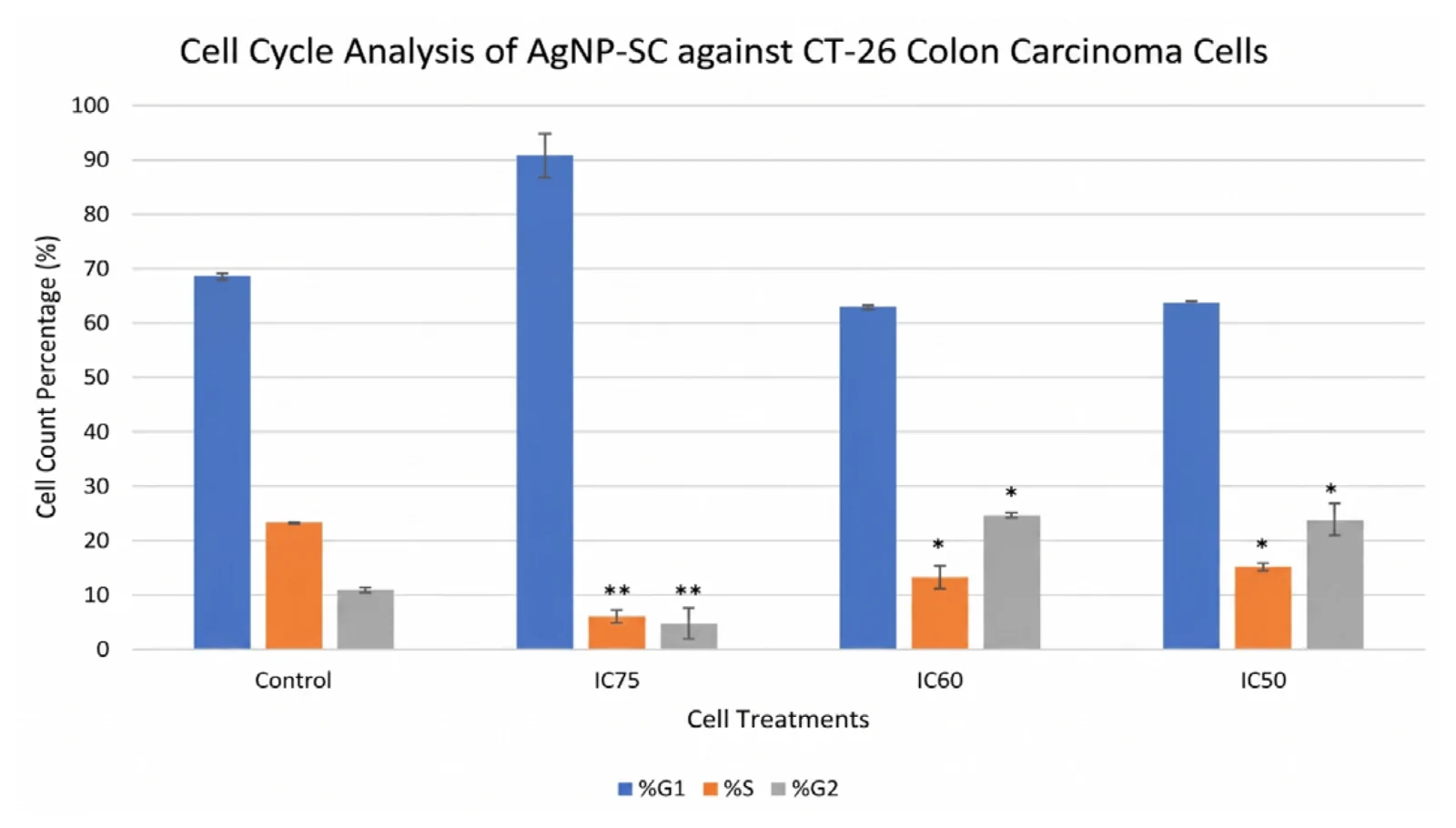
Cell cycle analysis of CT26 treated with IC_75_, IC_60_ and IC_50_ concentrations of AgNP-SC for 24 h. *Statistical analysis showed that the concentration is significantly different from the control *P* < 0.05.

**Figure 5:**
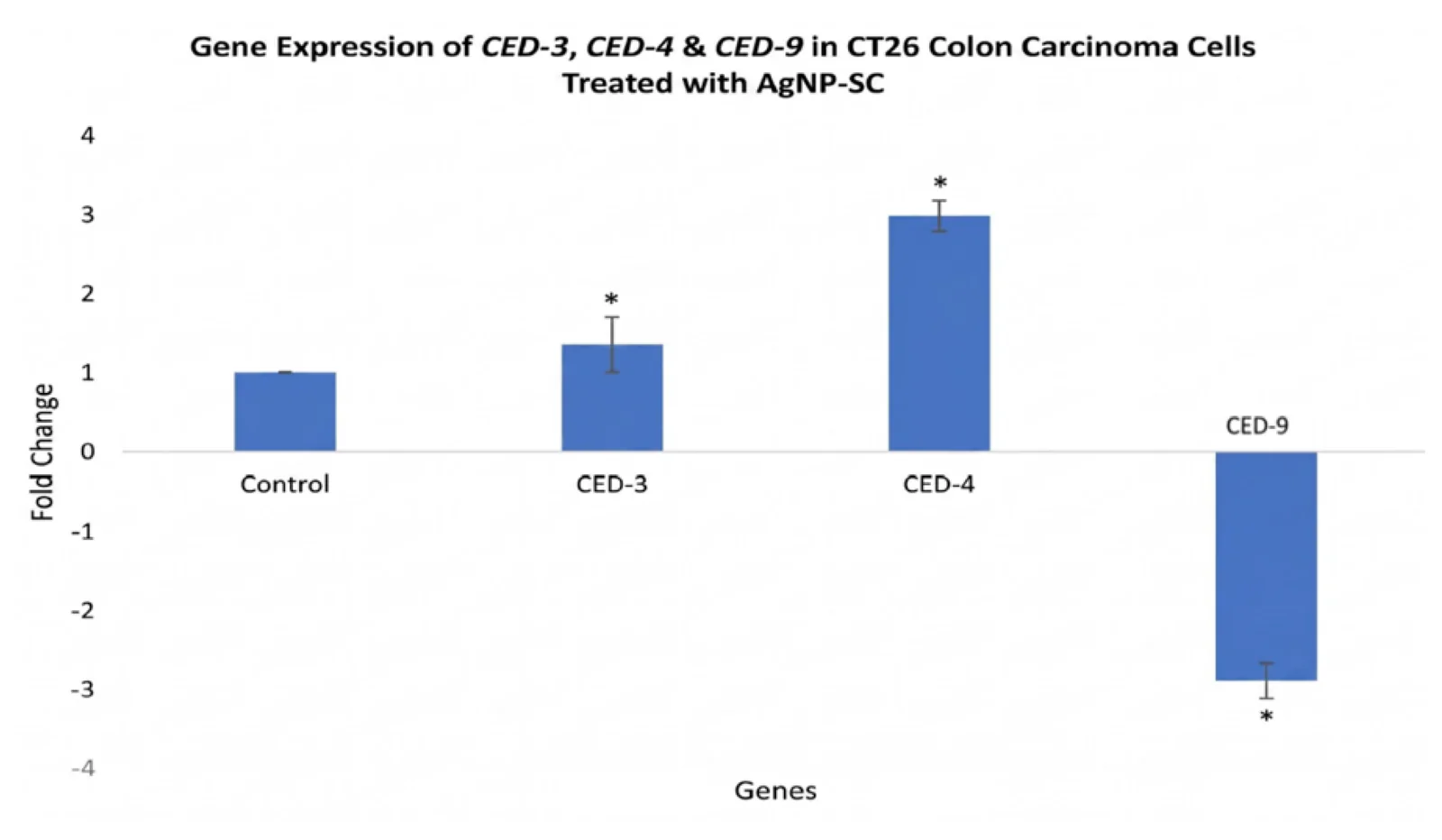
Relative expression of *CED-3, CED-4*, and *CED-9* genes in CT26 colon carcinoma cells following AgNP-SC treatment. Data are presented as mean ± SD. *Indicates a statistically significant difference compared with the control group (*P* < 0.05).
